# A Systematic Review and Meta‐Analysis of Randomized Controlled Trials on Fascial Defect Closure Versus Bridged Repair in Laparoscopic Ventral Hernia Mesh Repair

**DOI:** 10.1002/wjs.70338

**Published:** 2026-03-30

**Authors:** Moadh Hwessa, Wajeeh Ullah Mahmood, Omar Lubbad, Krishna K. Singh, Goldie Khera, Muhammad S. Sajid

**Affiliations:** ^1^ Brighton and Sussex Medical School Brighton UK; ^2^ Department of Gastrointestinal Surgery Royal Sussex County Hospital Brighton UK

**Keywords:** fascial defect closure, laparoscopic ventral hernia mesh repair, ventral hernia

## Abstract

**Introduction:**

This study compares the outcomes in patients undergoing ventral hernia repair using intraperitoneal underlay mesh (IPUM) without facial defect closure versus intraperitoneal underlay mesh following fascial defect closure (IPUM+).

**Methods:**

To search for randomized controlled trials comparing outcomes of patients with ventral hernias who were managed surgically with either IPUM or IPUM+, standard medical databases such as MEDLINE, Embase, PubMed, and Cochrane Library were used, covering studies published up to and including September 2025. The meta‐analysis was performed with a random effect model analysis, and all data was analyzed using Review Manager Software 5.4.

**Results:**

Five randomized controlled trials (*n* = 549) were included, involving adult patients who underwent laparoscopic ventral hernia repair with IPUM or IPUM+. The pooled analysis showed no significant difference in hernia recurrence (Risk Ratio [RR]: 0.82, 95% CI: [0.29, 2.27], *p* = 0.70), seroma formation (RR: 0.78, 95% CI: [0.32, 1.88], *p* = 0.58), operating times (Standard Mean Difference [SMD]: 0.26, 95% CI [−0.17, 0.69], *p* = 0.23) and pain scores (SMD: 0.26, 95% CI [−0.17, 0.69], *p* = 0.23) between the two approaches.

**Conclusion:**

IPUM and IPUM+ are both associated with similar postoperative morbidities for the treatment of ventral hernia repair.

## Graphical abstract

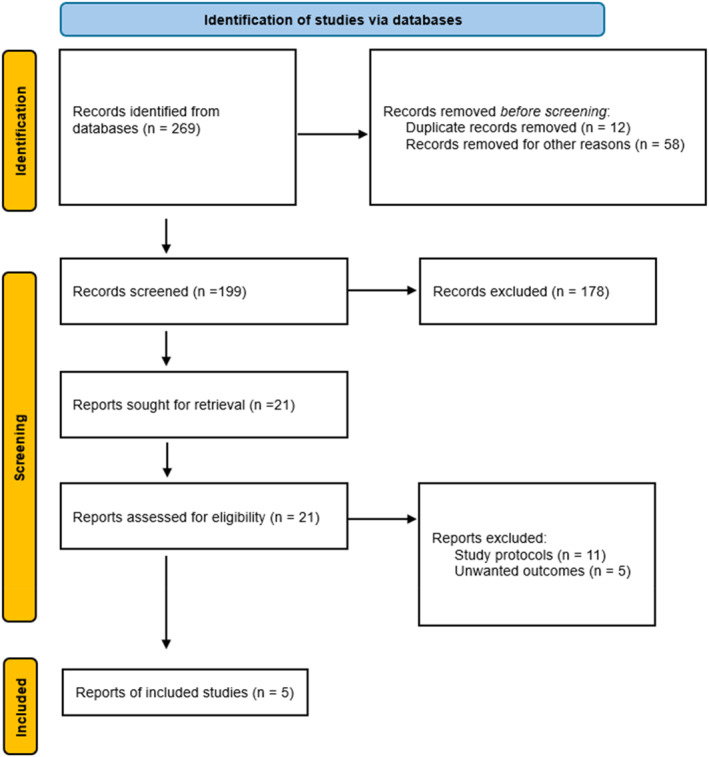

## 1 Introduction

Over 700,000 ventral hernia repairs are performed worldwide annually [1]. Patients with ventral hernias can often present with symptoms of discomfort and pain which can significantly impact their quality of life (QoL) [2]. If left untreated, there are risks of bowel obstruction, incarceration and strangulation [3]. Laparoscopic ventral hernia mesh repair (LVHMR) has emerged as a cornerstone in the management of anterior abdominal wall hernias, encompassing both primary and incisional defects [4]. Over the past 2 decades, the adoption of LVHMR has been driven by its demonstrable advantages over open repair techniques, including reduced postoperative wound morbidity, shorter hospitalization, expedited recovery, and improved patient‐reported outcomes [5]. Although robotic platforms have expanded in ventral hernia surgery, the laparoscopic approach continues to account for a meaningful proportion of minimally invasive repairs in contemporary practice [6]. This supports the continued clinical relevance of optimizing laparoscopic technique, including whether defect closure should be routinely performed prior to mesh fixation. A central consideration in LVHMR is whether to adopt a bridged repair approach, wherein the mesh spans the defect without primary closure, commonly referred to as intraperitoneal underlay mesh (IPUM) or perform fascial defect closure (FDC), also known as IPUM+. FDC is theorized to restore the structural integrity of the abdominal wall, thereby mitigating the risk of bulging or incisional eventration, improving functional abdominal wall mechanics, and potentially lowering recurrence rates [7, 8]. In contrast, IPUM, while technically less demanding and potentially associated with lower immediate tension on the repair site, has been linked in some observational studies to higher incidences of seroma formation, bulging, and hernia recurrence [5].

Current recommendations from leading hernia societies, including the European Hernia Society (EHS), the Americas Hernia Society (AHS), and the International Endohernia Society, favor FDC where technically feasible [9]. These guidelines are informed by previous systematic reviews of observational studies, which collectively suggest that FDC is associated with reduced recurrence and postoperative seroma formation. Previous meta‐analyses by Tandon et al. [10] and He et al. [4] used a combination of observational studies, including cohort and comparative studies, and showed that FDC was beneficial in reducing the rate of adverse wound complications and seroma formation. However, the observational nature of this evidence, patient selection and follow‐up protocols limits the certainty and generalizability of these findings.

The objective of the present study is to conduct the most comprehensive systematic review and meta‐analysis, comprising exclusively of randomized controlled trials (RCTs), including a recently published RCT by Lindmark et al. [11], comparing IPUM and IPUM+ in LVHMR. By focusing only on high‐level evidence, this analysis aims to elucidate differences in recurrence risk, complication rates, functional outcomes, and patient‐centered measures, thereby informing both clinical practice and guideline development.

## 2 Material and Methods

This meta‐analysis was conducted in accordance with the preferred reporting items for a systematic review and meta‐analysis, through the Preferred Reporting Items for Systematic Reviews and Meta‐analyses (PRISMA) guidelines [12]. This study was prospectively registered on Prospero (ID: 1179571) and a protocol was not prepared.

### 2.1 Data Sources and Search Strategy

For this systematic review and meta‐analysis, major medical databases including MEDLINE, EMBASE, PubMed, and the Cochrane Library were searched for eligible RCTs. The search covered publications from database inception up to and including September 2025 and used the terms “fascial defect closure versus bridged repair in laparoscopic ventral hernia mesh repair” and “hernia repair with and without closure.” Boolean operators (AND, OR, NOT) were applied to optimize the search strategy.

### 2.2 Study Selection

Only RCTs involving human participants that directly compared IPUM+ versus IPUM in patients undergoing LVHMR were included. Studies focusing on posterior hernias were excluded, as were quasi‐experimental designs, cohort studies, and case‐control studies. No limitations were placed on study location, language, or hospital setting. The primary outcomes of interest were seroma development, hernia recurrence, and postoperative pain measured by the visual analog scale (VAS).

### 2.3 Data Extraction

Data from the selected studies were extracted independently by two reviewers using a standardized meta‐analysis form, and a third reviewer checked the accuracy. No discrepancies arose between reviewers in either study selection or data extraction.

### 2.4 Statistical Analysis

Statistical analyses were carried out using Review Manager Version 5.4 [13] (Cochrane Collaboration). For binary outcomes, odds ratios (OR) with 95% confidence intervals (CIs) were calculated, while continuous variables were analyzed using the standardized mean difference (SMD) with 95% CIs. A random‐effects model [14, 15] was applied when pooling dichotomous outcomes. Heterogeneity was assessed using the *χ* ^2^ test (significance level *p* < 0.05) and quantified [16] with the *I* ^2^ statistic, where values ≤ 30% were considered to indicate low heterogeneity. When standard deviations were not reported, they were derived following Cochrane Collaboration recommendations [17]. RRs under the random‐effects model were estimated using the Mantel–Haenszel method [18]. Only RCTs that were clinically comparable and evaluated the same outcomes (e.g., seroma formation or duration of operation) were combined in the analysis. For sensitivity analysis, a continuity correction of 0.5 was added to each cell when no events were observed in either group, in line with the approach suggested by Deeks et al. [18]. This assumed equal variance across groups, although in some cases variance was estimated from reported ranges or *p* values. Pooled effect sizes were calculated based on inverse variance weighting. Forest plots were used to visually present results: squares indicated the precision of the estimates (reflecting sample size), and horizontal lines denoted 95% CIs. The methodological quality of included studies was appraised using the Cochrane risk of bias tool [19].

## 3 Results and Discussion

**FIGURE 1:**
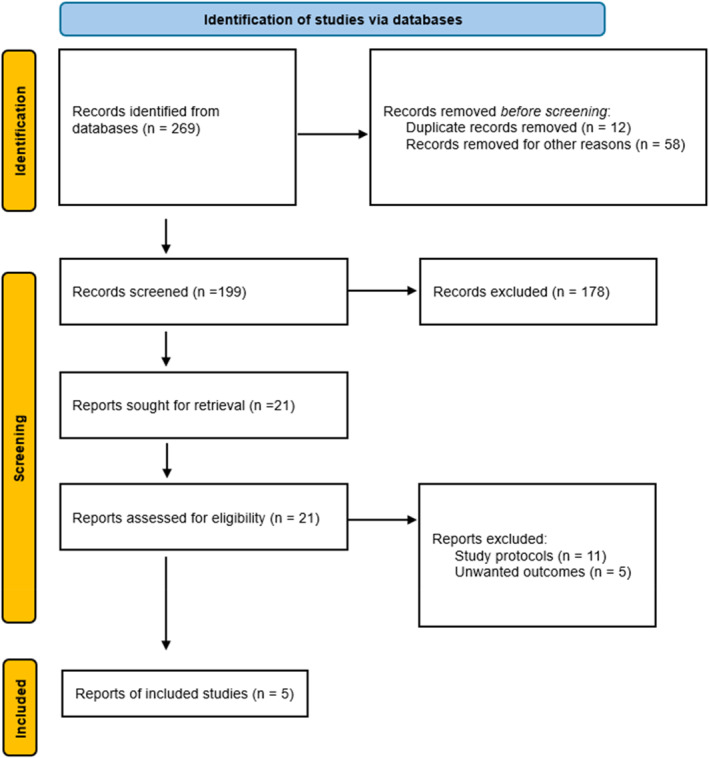
PRISMA flow chart explaining identification of studies via databases.

The primary database search led to two hundred and sixty‐nine studies, of which two hundred and sixty‐four were excluded after initial screening. The final review included five studies, as depicted in Figure 1.

### 3.1 Characteristics and Demographics of Included Studies

Five RCTs on 549 patients were included in our updated systematic review comparing patient outcomes between IPUM and IPUM+ in patients undergoing LVHMR. All surgeries were done via a laparoscopic approach and any that needed to be converted to open surgery, were excluded from the study. The trials included originated from Sweden [11, 20], the United States of America [21], Denmark [22] and Pakistan [23]. Studies were published between 2020 and 2025. Table 1 outlines the characteristics of the studies used and Table 2 highlights the protocol used in each study [20, 21, 22, 23].

**TABLE 1:** Characteristics of included studies.

Trial	Country	Patients	Age in years	Body mass index	Sex F:M	Type of hernia Primary: Incisional	Follow‐up in weeks	Trial running time
Ali 2020 [20]	Sweden						52 weeks	September 2017 to May 2018
IPUM+		24	58 ± 5.5^a^	29 ± 2.97^a^	12:13	9:16		
IPUM		24	56 ± 4^a^	30 ± 5.2^a^	9:16	7:18		
Bernardi 2020 [21]	USA						104 weeks	March 2015 to May 2017
IPUM+		64	50.1 ± 11.4	30 ± 4.4	41:23	13:51		
IPUM		65	49.1 ± 12	30.6 ± 3.6	41:24	16:49		
Christoffersen 2020 [22]	Denmark						104 weeks	December 2013 to August 2016
IPUM+		40	54 ± 11.25^a^	24 ± 12.25	18:22	35:5		
IPUM		40	55 ± 11.75^a^	27 ± 5.25	11:29	34:6		
Khan 2022 [23]	Pakistan						2 weeks	October 2019 to May 2020
IPUM+		50	43.92 ± 10.77^b^	33.26 ± 4.39	28:22	50:0		
IPUM		50			37:13	50:0		
Lindmark 2025 [10]	Sweden						52 weeks	November 2015 to September 2020
IPUM+		97	57 ± 12	31 ± 5	41:58	50:37		
IPUM		95	55 ± 13	32 ± 6	41:59	49:39		

^a^ SD estimated from range.

^b^ Combined mean from both groups.

**TABLE 2:** Treatment protocol adopted in included studies.

Trial	IPUM+	IPUM
Ali 2020 [20]	Midline ventral hernia defect of 3–10 cm Prophylactic antibiotics were given at the time of induction of anesthesia One 12‐mm and two 5‐mm ports were used for laparoscopic approach Hernia defect was closed with a 2–0 polydioxanone suture Specific brand of mesh used was not reported Intraperitoneal onlay mesh was used The edges of the fascial defect were fixed with the remaining tacks using double crown technique	Midline ventral hernia with a defect of 3–10 cm Prophylactic antibiotics were given at the time of induction of anesthesia One 12‐mm and two 5‐mm ports were used for laparoscopic approach Specific brand of mesh used was not reported Intraperitoneal onlay mesh was used
Bernardi 2020 [21]	Laparoscopic ventral hernia defect measuring between 3 and 10 cm in width Patient underwent pre‐operative skin preparation and given antibiotics Intraperitoneal onlay mesh was used The mesh is secured with four 0‐polydioxanone positioning sutures and tacked with a double crown of permanent tacks Defect was closed before mesh placement	Laparoscopic ventral hernia defect measuring between 3 and 10 cm in width Patient underwent pre‐operative skin preparation and given antibiotics Intraperitoneal onlay mesh was used The mesh is secured with four 0‐polydioxanone positioning sutures and tacked with a double crown of permanent tacks
Christoffersen 2020 [22]	Laparoscopic umbilical or epigastric hernia measuring 2–6 cm A 12‐mm trocar was placed along the left side lateral to the mid‐clavicular line under the left lower costal margin (Palmer's point). Additionally, one 5‐mm trocar and one 12‐mm trocar were placed in a vertical line downwards The hernia sac was not excised One liter of isotonic saline is infused during the surgical procedure independently of the patient's weight A physiomesh (Ethicon, NJ, USA) is placed with at least a 5‐cm overlap (measured on the non‐sutured gap) of the gap and fixated with double‐crown technique (Protack; Covidien, CN, USA). Defect was closed before intraperitoneal onlay mesh fixation using non‐absorbable interrupted sutures (Ethibond) Fascial trocar site defects > 5 mm are closed with polydioxanone PDS interrupted sutures, and skin is closed with nylon 3–0, single stitches.	Laparoscopic umbilical or epigastric hernia measuring 2–6 cm A12‐mm trocar was placed along the left side lateral to the mid‐clavicular line under the left lower costal margin (Palmer's point). Additionally, ing one 5‐mm trocar and one 12‐mm trocar were placed in a vertical line downwards The hernia sac was not excised One liter of isotonic saline is infused during the surgical procedure independently of the patient's weight A physiomesh (Ethicon, NJ, USA) is placed with at least a 5‐cm overlap (measured on the non‐sutured gap) of the gap and fixated with double‐crown technique (Protack; Covidien, CN, USA) Intraperitoneal onlay mesh fixation Fascial trocar site defects > 5 mm are closed with polydioxanone PDS interrupted sutures, and skin is closed with nylon 3–0, single stitches
Khan 2022 [23]	Laparoscopic primary repair of para‐umbilical hernia by closure of defect with non‐absorbable suture prior to mesh placement Mesh of suitable size with 4 cardinal sutures placed 1 cm from the edges was inserted inside the abdominal cavity and placed in the proper position to overlap 5 cm in all directions of the parietal defect using cardinal sutures Defect was closed using non‐absorbable sutures before mesh placement	Laparoscopic repair of para‐umbilical hernia without primary closure of the defect Mesh of suitable size with 4 cardinal sutures placed 1 cm from the edges was inserted inside the abdominal cavity and placed in the proper position to overlap 5 cm in all directions of the parietal defect using cardinal sutures
Lindmark 2025 [10]	Primary or incisional midline hernia with a transverse diameter ≥ 2 to ≤ 8 cm Surgery was performed under general anesthesia with a single dose of antibiotics (Eusaprim 160 mg + 800 mg and Metronidazol 1500 mg) administered preoperatively The prosthetic mesh used in this study was Symbotex (Medtronic, Minneapolis, MN, USA) which was fixed with a resorbable tacker Securestrap (SS; Ethicon US LLC, Ethicon Inc., Somerville, NJ, USA) in a double‐crown manner Port sites > 5 mm were closed with slowly resorbable polydioxiane (PDS 2.0, (SS; Ethicon US LLC, Ethicon Inc., Somerville, NJ, USA)) Defect was closed using a running suture of self‐retaining non‐resorbable V‐loc (2.0 or 0) (Medtronic, Minneapolis, MN, USA)	Primary or incisional midline hernia with a transverse diameter ≥ 2 to ≤ 8 cm Surgery was performed under general anesthesia with a single dose of antibiotics (Eusaprim 160 mg + 800 mg and Metronidazol 1500 mg) administered preoperatively The prosthetic mesh used in this study was Symbotex (Medtronic, Minneapolis, MN, USA) which was fixed with a resorbable tacker Securestrap (SS; Ethicon US LLC, Ethicon Inc., Somerville, NJ, USA) in a double‐crown manner Port sites > 5 mm were closed with slowly resorbable polydioxiane (PDS 2.0, (SS; Ethicon US LLC, Ethicon Inc., Somerville, NJ, USA)).

### 3.2 Methodological Quality of Included Studies

The methodological quality of the included RCTs is shown in Table 3 [20, 21, 22, 23]. Randomization was achieved through either simple randomization using a lottery method, block randomization and computer generation. Concealment was reported in three studies [11, 20, 22]. Double‐blinding was reported in four studies [11, 20, 21, 22], with one being single‐blinded [23]. Two studies followed an intentional‐to‐treat protocol [20, 22] and all five reported using a power calculation to determine the confidence interval [11, 20, 21, 22, 23].

**TABLE 3:** Quality assessment of included studies.

Trial	Randomization technique	Power calculation	Blinding	ITT^a^	Concealment	Inclusion criteria	Exclusion criteria
Ali 2020 [20]	Computerized randomization	Yes	Double‐blinded	Yes	Yes	Yes	Yes
Bernardi 2020 [21]	Computer‐generated block randomization	Yes	Double‐blinded	NR^b^	No	Yes	Yes
Christoffersen 2020 [22]	Block randomization	Yes	Double‐blinded	Yes	Yes	Yes	Yes
Khan 2022 [23]	Simple randomization (lottery method)	Yes	Single‐blinding	NR^b^	NR^b^	Yes	Yes
Lindmark 2025 [10]	Computer‐generated randomization	Yes	Double‐blinded	No	Yes	Yes	Yes

^a^ ITT = Intention to treat.

^b^ NR = Not reported.

## 4 Outcomes

### 4.1 Hernia Recurrence

**FIGURE 2:**
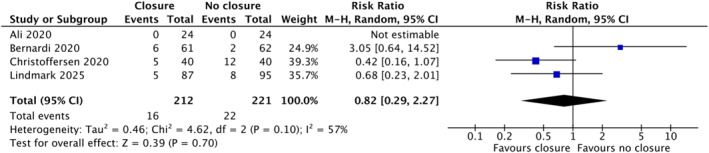
Forest plot showing hernia recurrence rate represented in risk ratio and 95% confidence interval.

Four studies reported hernia recurrence with a total of 439 patients. In the random effects model analysis, the incidence of hernia recurrence was statistically similar (RR 0.82, 95%, Cl (0.29, 2.27), *Z* = 0.39, *p* = 0.70) in both groups. However, there was significant heterogeneity among included studies (Tau^2^ = 0.46; *χ* ^2^ = 4.60, df = 2; *p* = 0.01; *I* ^2^ = 57%; Figure 2) [11, 20, 21, 22].

### 4.2 Seroma Formation

**FIGURE 3:**
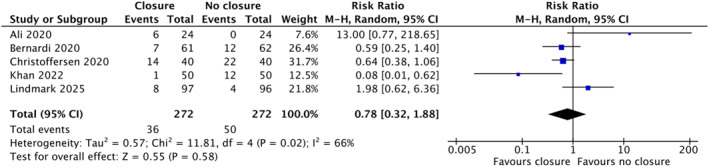
Forest plot showing seroma formation represented in risk ratio and 95% confidence interval.

Across five studies involving a total of 550 patients, seroma formation was reported as an outcome. Pooled analysis using a random‐effects model demonstrated no significant difference between groups (RR 0.78, 95% CI 0.32–1.88; *Z* = 0.55; *p* = 0.58). However, there was significant heterogeneity among the included studies (Tau^2^ = 0.57; *χ* ^2^ = 11.81, df = 4; *p* = 0.02; *I* ^2^ = 66%; Figure 3) [11, 20, 21, 22, 23].

### 4.3 Duration of Operation

**FIGURE 4:**
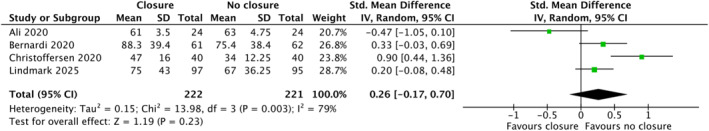
Forest plot showing duration of operation represented in standard mean difference and 95% confidence interval.

An analysis of four studies comprising 449 patients evaluated the duration of the operation. Pooled analysis using a random‐effects model showed no significant difference between groups (SMD 0.26, 95% CI −0.17 to 0.69; *Z* = 1.20; *p* = 0.23). However, substantial heterogeneity was observed among the included studies (Tau^2^ = 0.15; *χ* ^2^ = 13.98, df = 3; *p* = 0.003; *I* ^2^ = 79%; Figure 4) [11, 20, 21, 22].

### 4.4 Pain Score

**FIGURE 5:**
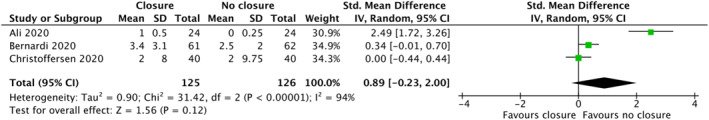
Forest plot showing pain scores represented in standard mean difference and 95% confidence interval.

Three studies reported collecting pain scores with a total of 257 patients. In the random effects model analysis, the pooled analysis was statistically similar (SMD: 0.89, 95%, Cl [−0.22, 1.99], *Z* = 1.57, *p* = 0.12) in both groups. However, there was significant heterogeneity among included studies (Tau^2^ = 0.88; *χ* ^2^ = 31.45, df = 2; *p* = < 0.00001; *I* ^2^ = 94%; Figure 5) [20, 21, 22].

## 5 Discussion

This is the most up‐to‐date systematic review and meta‐analysis comparing IPUM with IPUM+ in LVHMR. We analyzed 5 RCTs involving 549 patients (275 who underwent IPUM+ and 274 who underwent IPUM). Our pooled analysis that there was no significant difference in hernia recurrence, seroma formation, operation times and pain scores in patients undergoing LVHMR between IPUM or IPUM + before mesh fixation.

### 5.1 Strengths

Being composed exclusively of RCTs strengthens this study, as randomization minimizes confounding factors and allows differences in outcomes to be confidently attributed to the intervention rather than external variables [24]. Other study designs, such as case series or observational studies used by Tandon et al. [10] and He et al. [4], are more vulnerable to confounding and selection bias. Restricting to RCTs strengthens the overall level of confidence, enhances the comparability of included studies, and ultimately increases the clinical relevance of the conclusions drawn [25]. Secondly, with a greater sample size, the statistical power of the analysis, reduces the risk of Type II errors. This allows for more precise estimates of treatment effects and enables the findings to be more generalizable and less likely to be due to chance, which strengthens the external validity of the results [26].

A key strength of this study is its low risk of bias, as summarized in Table 3. The methodology demonstrates appropriate randomization, allocation concealment, and blinding of outcome assessment, reducing the likelihood of systematic errors. This enhances the internal validity of the findings and increases confidence that the observed effects are attributable to the intentions rather than to methodological flaws. Finally, the inclusion of recent RCTs ensures that the evidence reflects contemporary surgical practice, including current mesh technologies fixation methods, and perioperative protocols. This strengthens the clinical relevance of the findings and supports their applicability to modern surgical settings.

### 5.2 Clinical Implications

In this updated RCT‐only meta‐analysis, IPUM+ during LVHMR was not associated with statistically significant differences in recurrence, seroma formation, operative duration, or postoperative pain compared with IPUM. From a clinical perspective, these findings support a tailored operative strategy in which the decision to close the defect is guided by technical feasibility, defect characteristics, and patient‐level risk rather than an assumption of uniform benefit. Where primary closure can be achieved without undue tension, IPUM+ may still be preferred to restore abdominal wall continuity and potentially reduce postoperative eventration and improve functional outcomes; conversely, IPUM remains a reasonable option when closure is technically prohibitive or would impose excessive tension, particularly in larger defects (> 4 cm per EHS & AHS classification) or complex abdominal wall anatomy [9, 27]. Moreover, an absence of statistically significant differences should not be interpreted as evidence of equivalence across all clinical contexts; rather, it reflects the limitations of the current RCT evidence base, including modest cumulative sample size, imprecision around effect estimates, and heterogeneity in operative technique and outcome ascertainment.

### 5.3 Future Directions

Future research should focus on pragmatic and adequately powered RCTs with longer follow‐up to capture late outcomes, as hernia recurrence and mesh‐related complications may manifest beyond the time horizons reported in several included trials. Standardization is required across trials for core outcome definitions and measurement: recurrence should be assessed using pre‐defined criteria with consistent clinical and/or imaging confirmation; seroma should be reported using clinically meaningful thresholds (e.g., symptomatic, intervention‐requiring, or persistent seroma) rather than incidental radiological findings; and patient‐centered outcomes should include validated QoL instruments and functional abdominal wall assessments, in addition to pain trajectories. Given the observed heterogeneity, trials should prespecify subgroup analyses by defect size, hernia type (primary vs. incisional), BMI, closure method, mesh characteristics. Finally, incorporation of health‐economic analyses would strengthen the relevance of future evidence for guideline development and service planning.

### 5.4 Comparison

There are many comparisons from this up‐to‐date meta‐analysis to the prior RCT‐only meta‐analyses. In 2022, Tryliskyy et al. [8] conducted a systematic review in 2022, which included 3 RCTs, confirming that both techniques have equal safety profiles but found there was no difference detected in the risk of hernia recurrence, clinical or radiological eventration and seroma formation, contradicting prior evidence [4, 8, 10]. An updated systematic review on this topic, conducted by Jeong et al. [28] included 5 RCTs, however, used a study by Ahonen‐Siirtola et al. [29] in which one of the arms was a hybrid approach, rather than directly comparing IPUM+ and IPUM. This limits the validity of its conclusion that IPUM+ significantly reduced seroma formation, as it introduces potential confounding factors. However, both Tryliskyy et al. and Jeong et al., concluded that high‐quality trials are needed to provide a more robust overview of whether IPUM+ has any benefit to LVHMR [8, 28].

### 5.5 Limitations

This study has multiple limitations that warrant consideration. Primarily, the modest number of RCTs included limits the generalizability of our findings. Furthermore, clinical heterogeneity related to patient and hernia characteristics, operative techniques, follow‐up duration, and outcome definitions varied between clinical and radiological detection, as highlighted by Tables 1 and 2. Furthermore, variability was seen in the type of sutures used for closure, most notably, Lindmark et al. using V‐Loc [30] suture as opposed to polydioxanone [31] and Ethibond [32] used in the other studies [11]. This highlights a likely cause for the heterogeneity between the studies and, critically affects the generalizability of the results found. In Table 3, details regarding whether the study followed an intention‐to‐treat protocol or allocation concealment was not clearly defined or reported and as a result, there is a risk of reporting and selection bias. In addition, the relatively short follow‐up period may limit the ability to detect late complications such as long‐term recurrence, potentially underestimating adverse outcomes and restricting conclusions regarding the durability of the intervention. Finally, our pooled analysis demonstrates that there is a high statistical heterogeneity across all the studies and their outcomes, hindering the accuracy and generalizability of the results.

## 6 Conclusion

In LVHMR, both arms have comparable short to mid‐term outcomes for recurrence, seroma formation, operative duration, and postoperative pain in LVHMR based on currently available randomized evidence. However, the certainty of this is limited by imprecision (wide confidence intervals), substantial heterogeneity across outcomes, variation in operative protocols and outcome definitions, and, critically, follow‐up durations that may be insufficient to detect late recurrence, eventration, and longer‐term mesh‐related complications. Accordingly, additional RCTs remain warranted, but the priority is not simply increasing trial count; rather, the field requires methodologically rigorous studies with longer follow‐up, harmonized outcome reporting, and stratification by clinically relevant factors (e.g., defect size, BMI, hernia type, mesh fixation strategy) to determine whether clinically meaningful differences emerge over time or within specific subgroups.

## Author Contributions

**Moadh Hwessa:** conceptualization, writing – original draft, methodology, software, formal analysis, writing – review and editing, investigation, funding acquisition, validation, visualization, project administration, data curation. **Wajeeh Ullah Mahmood:** software, methodology, writing – review and editing, formal analysis. **Omar Lubbad:** validation, software, writing – review and editing, methodology. **Krishna K. Singh:** software, formal analysis, project administration, writing – review and editing, supervision. **Goldie Khera:** supervision, visualization, resources, project administration, writing – review and editing. **Muhammad S. Sajid:** investigation, supervision, software, writing – review and editing, conceptualization, methodology, formal analysis.

## Funding

The authors have nothing to report.

## Conflicts of Interest

The authors declare no conflicts of interest.

## Data Availability

Data available upon request.
